# Axillary metastasis as the first manifestation of male breast cancer: a case report

**DOI:** 10.1186/1757-1626-1-285

**Published:** 2008-10-30

**Authors:** Guo-Li Gu, Shi-Lin Wang, Xue-Ming Wei, Li Ren, Fu-Xian Zou

**Affiliations:** 1 Department of General Surgery, the General Hospital of Chinese PLA Air force, Beijing City, 100142, PR China; 2 Department of Pathology, the General Hospital of Chinese PLA Air force, Beijing City, 100142, PR China; 3 Surgical Department of the Affiliated Hospital, Jiangxi University of Science and Technology, Ganzhou City, Jiangxi Province 341000, PR China

## Abstract

**Background:**

Breast cancer is very rare in men, and the occurrence of occult breast cancer which present axillary metastasis as the first manifestation is even rarer in men.

**Case presentation:**

We report a 72-year-old male Han-Chinese patient who presented axillary metastasis as the first manifestation of breast cancer and got correctly diagnoses by histological examination. He underwent modified radical mastectomy and axillary dissection on 11 Apr 2006. The histopathologic examination showed that no tumor focus was found in his breast tissue, but two out of fifteen of axillary lymph nodes were invaded by infiltrating ductal carcinoma. The IHC stain showed that estrogen receptor (ER) and progestin receptor (PR) were negative, Human epidermal receptor (HER-2) oncoprotein (+++), P53 protein expressed (+++), Bcl-2 oncoprotein (+++), nm23 protein (++), proliferating cell nuclear antigen (PCNA) (+++) and multidrug-resistance protein (MRP) (++). After operation, he did not receive endocrine therapy, chemotherapy and radiotherapy because of his senility. He is alive without any residual or metastasis disease 29 months after being diagnosed.

**Conclusion:**

This is the first case in our hospital that presents axillary metastases as the first manifestation of male breast cancer.

## Background

Breast cancer is very rare in men, accounting for less than 1% of all breast cancers and less than 1% of all cancers in men [1,2]; and the occurrence of occult breast cancer is even rarer in men [3]. We report a 72-year-old male Han-Chinese patient with occult breast cancer who was diagnosed by histological examination and Immunohistochemical (IHC) stain. It is the first male case of our hospital that presents axillary metastasis as the first manifestation of occult breast cancer.

## Case presentation

**Figure 1:**
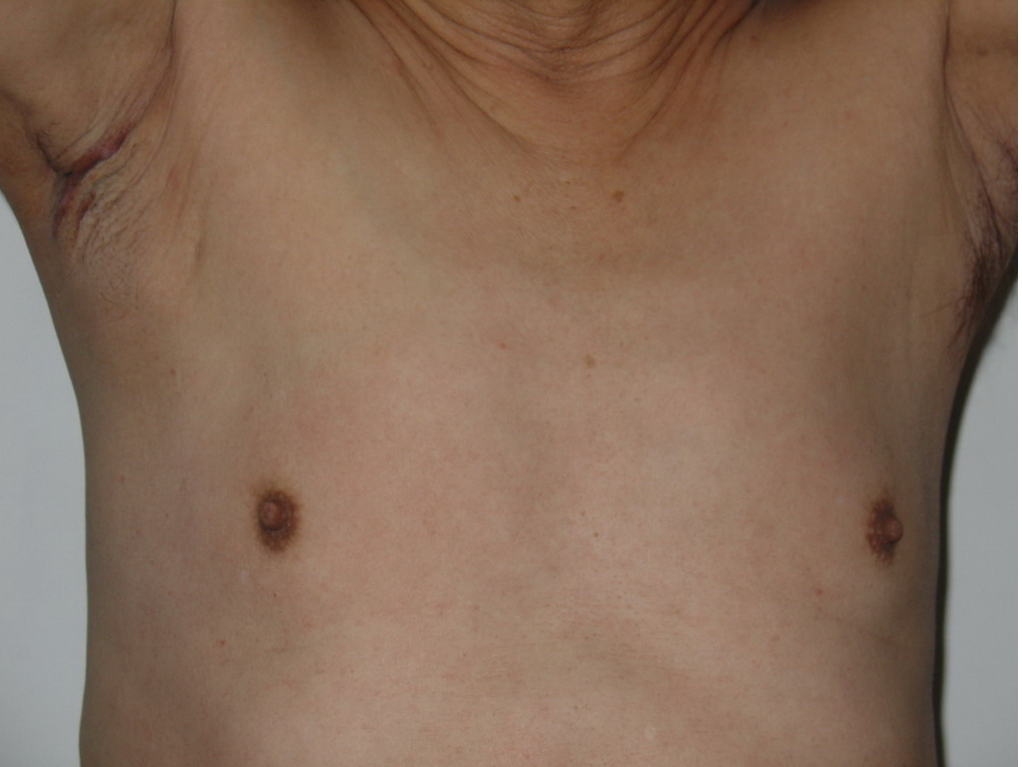
**External photography of patient's chest**. showing enlarged lymph node in the right axillary after biopsy, without palpable breast mass.

**Figure 2:**
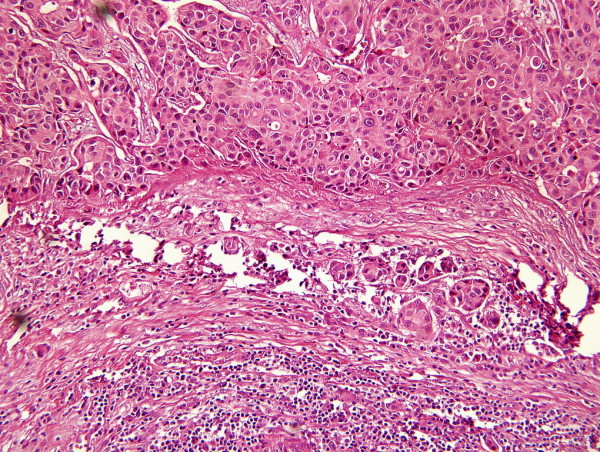
**Metastatic IDC in axillary lymph node**. the structure of lymph node which was invaded by metastatic IDC has been destroyed. Big, relatively round tumor cells can be seen invading the lymph node tissue (HE stain, original magnification ×200).

**Figure 3:**
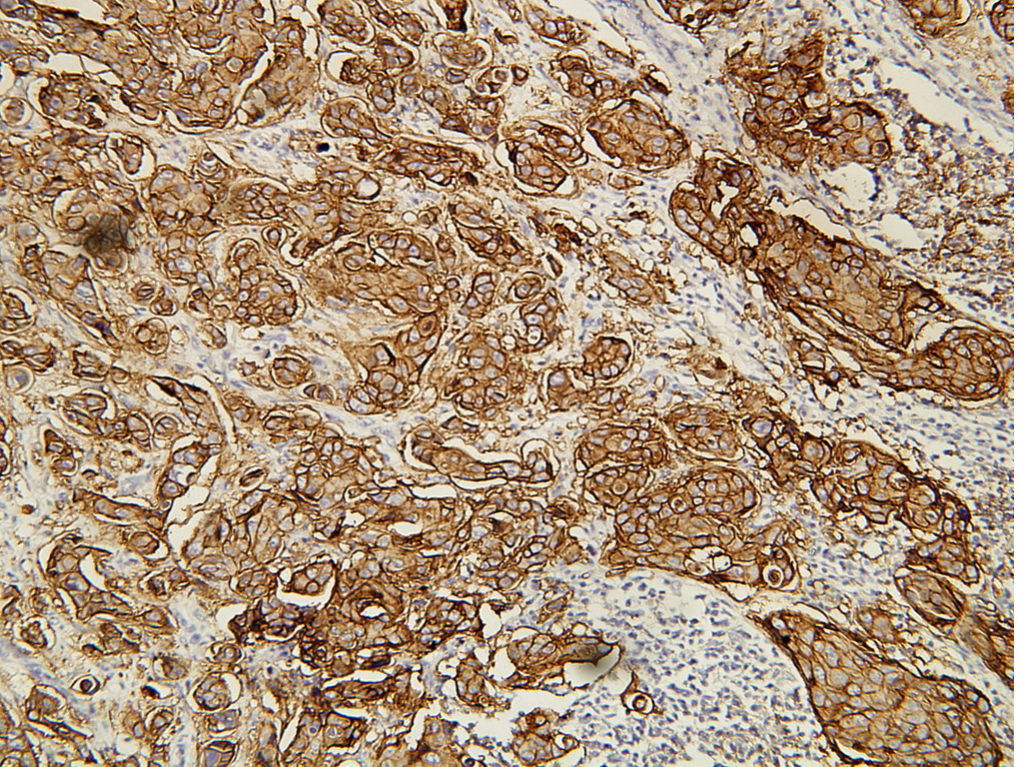
**IHC stain of HER-2 oncoprotein**. Showing positive expression of HER-2 oncoprotein on carcinoma cell membrane. (SP ×200).

**Figure 4:**
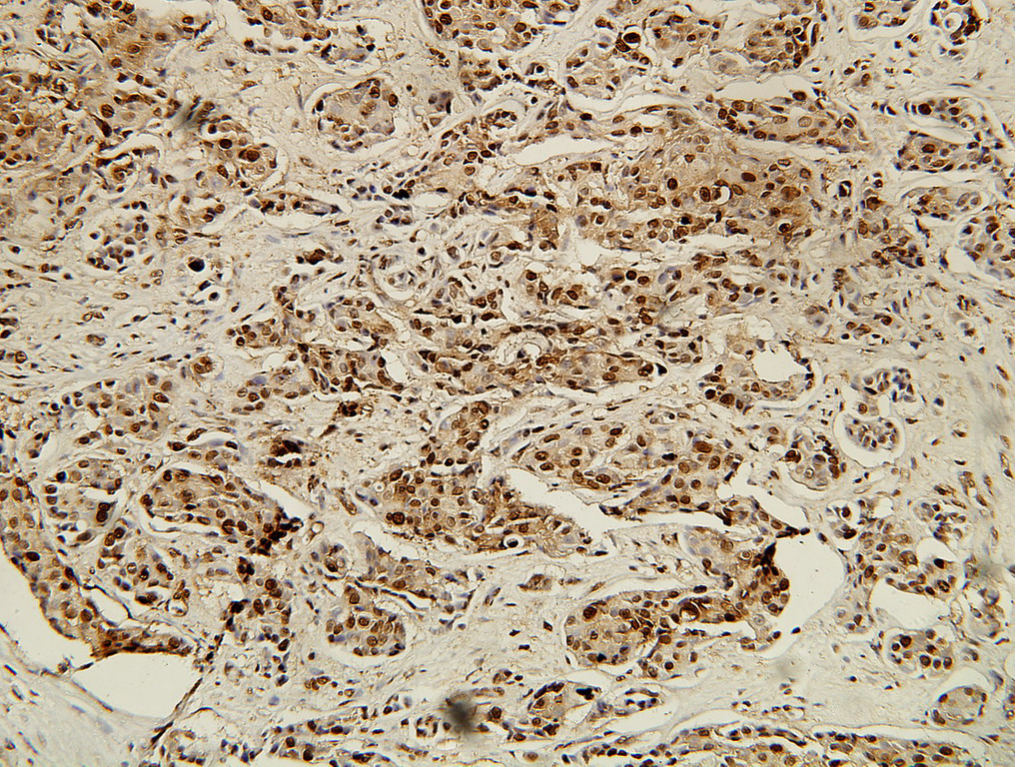
**IHC stain of PCNA**. Showing positive expression of PCNA in carcinoma cell nuclei. (SP ×200).

**Figure 5:**
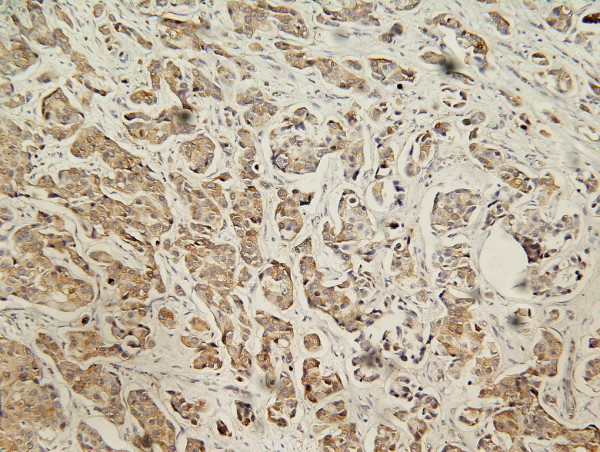
**IHC stain of Bcl-2 oncoprotein**. Showing positive expression of Bcl-2 oncoprotein in carcinoma cell cytoplasm. (SP ×200).

**Figure 6:**
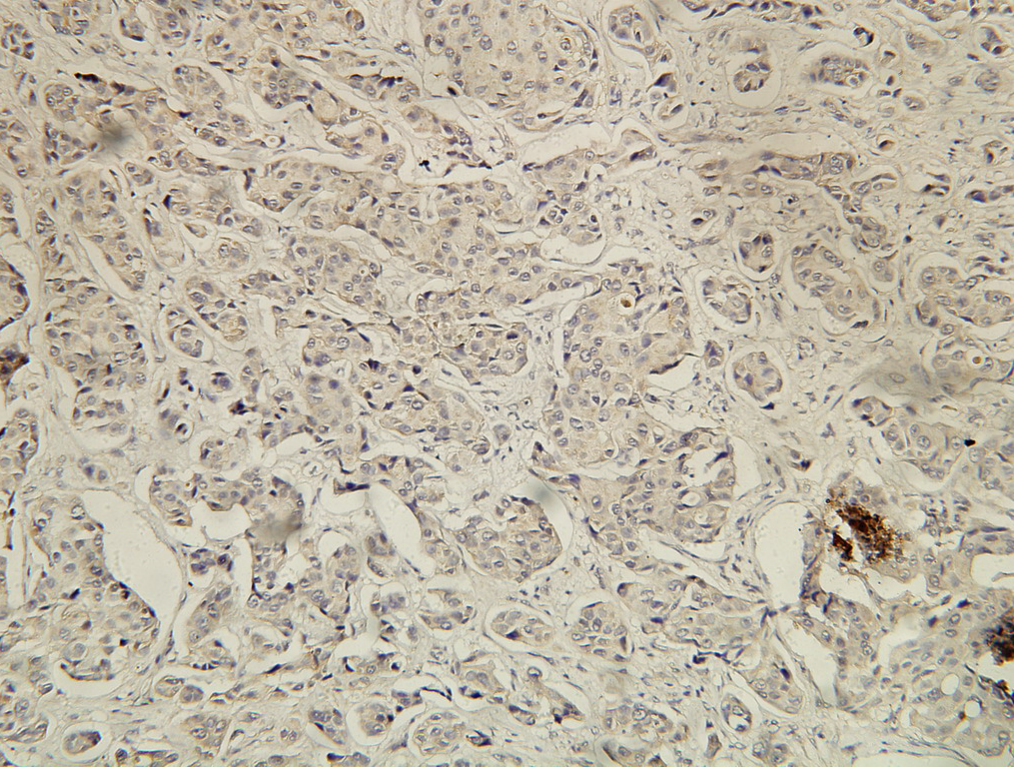
**IHC stain of nm23 protein**. Showing positive expression of nm23 protein in carcinoma cell cytoplasm. (SP ×200).

**Figure 7:**
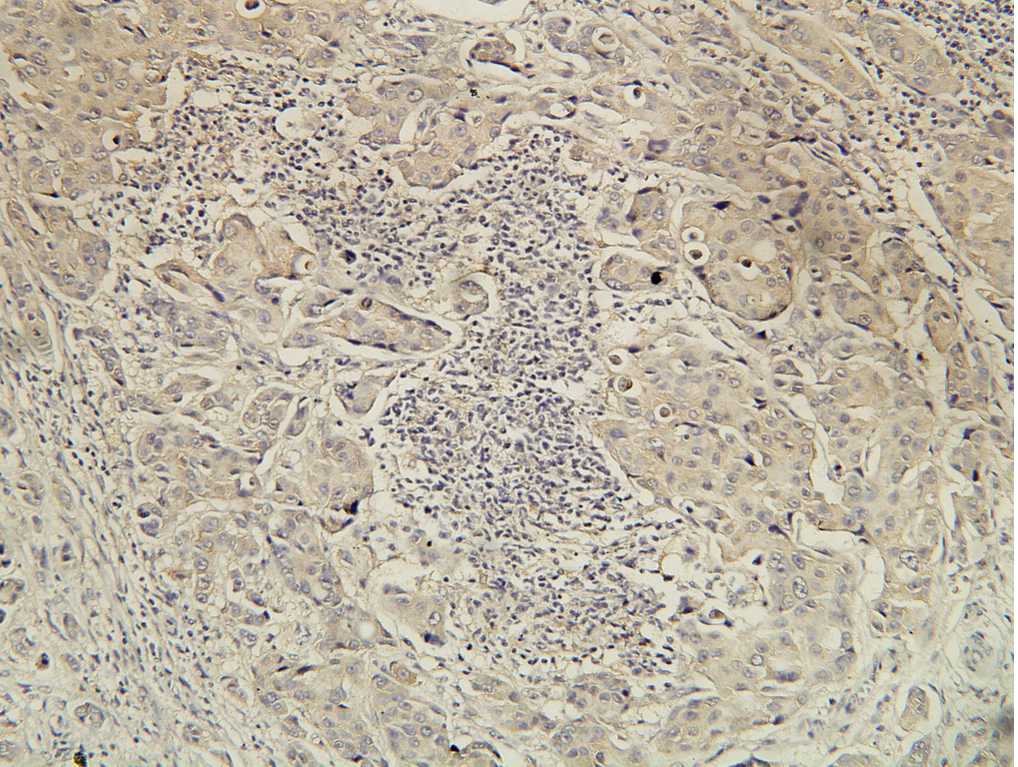
**IHC stain of MRP**. Showing positive expression of MRP in carcinoma cell cytoplasm. (SP ×200).

**Figure 8:**
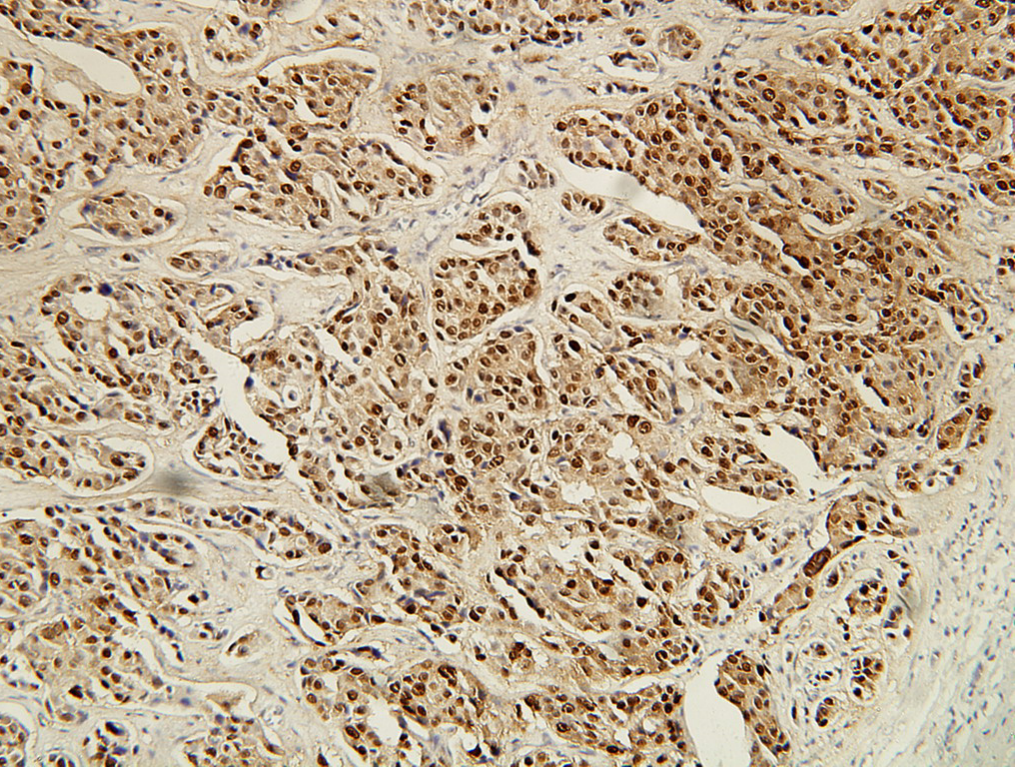
**IHC stain of P53 protein**. Showing positive expression of P53 protein in carcinoma cell nuclei. (SP ×200).

A 72-year-old male Han-Chinese patient presented with a painless and enlarged lymph node in the right axillary for about 6 months without any palpable breast mass (Figure 1). His history showed that no evidence of liver diseases; no medication had been taken, and in particular, there was no history of hormonal treatment. The axillary lymph node biopsy showed that it was invaded by infiltrating ductal carcinoma (IDC) which was in suspect metastasized from breast. The patient was kept in hospital and took a series of physical, X-ray and ultrasonic examinations. But no other focuses were found in body. The result of the hepatic function test was natural. The biopsy slice was circulated and opinions were sought from fellows of other hospitals. Then, the pathologists came to a conclusion that the male patient was a case with occult breast cancer. The patient underwent a modified radical mastectomy and axillary dissection on 11 April 2006. The histological examination of paraffin sections stained with hematoxylin and eosin (HE) revealed that no tumor focus was found in breast, but two out of fifteen of axillary lymph nodes were invaded by IDC. The histological examination showed the lymph tissue massively and diffusively infiltrated with big and relatively round tumor cells that are tightly cohesive and displayed round-to-ovoid nuclei and a thin rim of cytoplasm with an occasional intracytoplasmic lumen (Figure 2). The IHC stain (Figure 3, 4, 5, 6, 7, 8) showed estrogen receptor (ER) and progestin receptor (PR) were negative, P53 protein expressed (+++), proliferating cell nuclear antigen (PCNA) (+++), Bcl-2 oncoprotein (+++), nm23 protein (++), multidrug-resistance protein (MRP) (++), Human epidermal receptor (HER-2) oncoprotein (+++). This patient did not receive endocrine therapy, chemotherapy and radiotherapy after operation. He is alive without any residual or metastasis disease 29 months after being diagnosed; and he is still in follow-up.

## Discussion

Breast cancer is very rare in men. Epidemiological studies showed that the rate of male breast cancer is 0.5%–1%, in most western countries, and 6% or more in Tanzania and in Central Africa [1,2]. The reasons for this geographic variability remain unclear. Recent epidemiologic data suggested that the incidence of male breast cancer has been steadily increasing [4]. However, the pathogenesis of male breast cancer is still unclear. The epidemiologic risk factors may include prostate cancer and its endocrine therapy, gynecomastia, occupational exposures (e.g., electromagnetic fields, polycyclic aromatic hydrocarbons, and high temperatures), dietary factors (e.g., meat intake, fruit and vegetable consumption), and alcohol intake. Recently, the genetic factors got more attention by scientists [4,5]. The mutations of BRCA1, BRCA2 and MMR gene may play very important roles in the onset of the male breast cancer; and other genetic factors involved could include *AR *gene, *CHEK2 *gene, cytochrome P45017 (*CYP17*), the XXY karyotype (Klinefelter syndrome), and the PTEN tumor suppressor gene associated with Cowden syndrome, and so on. The *BRCA1 *and *BRCA2 *germ-line mutation is known associated with the hereditary breast cancer [6]; and the MMR germ-line mutation (especially *hMLH1*) for the hereditary nonpolyposis colorectal carcinoma (HNPCC). Some researchers regarded the breast cancer, especially the male breast cancer, as a part of the tumor spectrum of HNPCC, and thought the breast cancer might be an extracolonic manifestation of HNPCC [7]. Therefore, if a male patient with breast cancer is met with in clinic, we should attend not only to examine the breast focus, but also to inquire the patient about his family history and post history which is helpful for clinical diagnosis and therapy.

Occult breast cancer is even rarer in men. It usually presents lymph node metastasis of axilla, supraclavicular fossa and infraclavicular fossa as the first manifestation [8]. In our case, the patient presented axillary lymph node metastasis as the first manifestation. The most common causes of axillary malignant lymph nodes include lymphoma and metastasis from breast cancer, lung cancer, melanoma, and squamous cell cancer. So, we should give our attention to take relevant examination to find focus and avoid misdiagnosis, if we meet with similar patients in clinic. Research shows that it is in approximately 50% cases with occult breast cancer the cancer focus still can not be found in the breast specimen [9]. In our case, no tumor focus was found in breast specimen. The final diagnosis was made after many pathologists' consultation and a series of examinations for differential diagnosis. Hereby, the histological examination and IHC stain of metastasis focus is very important in the diagnosis of the occult breast cancer.

The currently recommended surgical therapy in clinic is the modified radical mastectomy with axillary dissection. It was reported that male breast carcinomas have a higher positive rate of hormone receptor than the female breast carcinomas, and so the adjuvant hormonal therapy is theoretically very promising [10]. In our case, although the tumor cells were negative for ER and PR, the expression results of PCNA, P53, Bcl-2, nm23, MRP and HER-2 protein were similar to other reports [1-4]. Because he was a septuagenarian, his families did not agree with him to undergo endocrine-therapy, chemotherapy and radiotherapy after operation. But the patient's prognosis seems well; he has survived with cancer-free for about 29 months after diagnosed, and he is still in follow-up.

## Consent

The consent was obtained from the patient for publication of this case report and accompanying images. We have obtained consent for publication in print and electronically from the patient.

## Competing interests

The authors declare that they have no competing interests. We all authors have seen and approved the submitted version of this manuscript. There was not a medical writer or editor involved in the generation of our manuscript. We are assured that the manuscript has not been published or submitted for publication elsewhere except as a brief abstract in the proceedings of a scientific meeting or symposium.

## Authors' contributions

Gu GL and Wang SL designed the research. Gu GL and Zou FX collected the clinical data and wrote the manuscript. Ren L collected the pathological data. Wang SL, Wei XM and Zou FX revised the manuscript.
